# Neck circumference as an independent indicator of visceral obesity in a Chinese population

**DOI:** 10.1186/s12944-018-0739-z

**Published:** 2018-04-17

**Authors:** Li Zhao, Guolan Huang, Fangzhen Xia, Qin Li, Bing Han, Yi Chen, Chi Chen, Dongping Lin, Ningjian Wang, Yingli Lu

**Affiliations:** 1 Institute and Department of Endocrinology and Metabolism, Shanghai Ninth People’s Hospital, Shanghai JiaoTong University School of Medicine, Shanghai, 200011 China; 2 Department of Endocrinology, Fengcheng Branch of Shanghai Ninth People’s Hospital, Shanghai JiaoTong University School of Medicine, Shanghai, 200011 China

**Keywords:** Neck circumference, Visceral obesity, Lipid metabolism

## Abstract

**Background:**

Neck circumference (NC) was reported to be associated with visceral obesity in some specific subjects. However, no studies have reported whether NC could identify visceral obesity in the general population. Here, we mainly aimed to explore whether NC is suitable to identify visceral obesity in the general population.

**Methods:**

Our data were from a cross-sectional survey on the prevalence of metabolic diseases and risk factors in East China from 2014 to 2015. A total of 9366 participants aged 18–93 were identified for analysis. Anthropometric indices, biochemical parameters and clinical characteristics were measured. The NC values were quartered according to sex. Spearman’s correlation coefficient was employed to test the correlations between different variables. Linear regression and logistic regression were conducted to explore the relationship of NC with visceral adiposity indices and visceral obesity.

**Results:**

Among the 9366 participants, 3938 (42.05%) were male and 5428 (57.95%) were female. NC had a positive correlation with the visceral adiposity indices, regardless of sex. In all quartiles of NC, in both men and women, as NC values increased, the values of all the fatness indices showed a tendency to increase (all *P* < 0.001). After full adjustment for demographic variables and metabolic factors, linear regression showed that NC was still associated with the fatness indices for visceral obesity (all *P* < 0.001). In addition, logistic analysis showed that a larger NC was associated with a higher risk of visceral obesity in both males (OR 32.34, 95% CI 24.02–43.53; *P* < 0.001) and females (OR 21.43, 95% CI 17.30–26.55; *P* < 0.001) after adjusting for potential confounding factors.

**Conclusion:**

NC can be a supplemental indicator for identifying visceral obesity in the general Chinese population.

## Background

According to the epidemiological data from the World Health Organization (WHO), there are more than half a billion adults with overweight or obesity [1]. Obesity, especially visceral obesity, is a well-known risk factor for many disorders, such as metabolic disease [2], hemodynamic, endothelial, inflammatory [3] and even physiological disorders. Thus, continuous monitoring and early detection of obesity can prevent the onset of its adverse outcomes. However, the distribution of adipose tissue plays a more important role than the amount in the development of these disorders [4].

Currently, the sophisticated methods to assess visceral adiposity include dual-energy X-ray absorptiometry (DEXA), computed tomography (CT) and magnetic resonance imaging (MRI) [5]. However, DEXA, CT and MRI are impractical for screening the general population, since they require expensive and specialized equipment, and patients are exposed to radiation. In this regard, many indices to estimate central or visceral obesity have been suggested. Among them, classical parameters such as waist circumference (WC), waist-to-hip ratio (WHR) and waist-to-height ratio (WHtR) are the most popular indices that are widely applied in clinical settings. More recent indices, including the visceral adiposity index (VAI), the lipid accumulation product (LAP), the abdominal volume index (AVI) and the conicity index (Cindex), which are calculated on the basis of simple data such as triglycerides (TG), high-density lipoprotein (HDL), body mass index (BMI), weight, height, WC, and hip circumference (HC), have also been introduced [6, 7]. In particular, the VAI has proven to be an indicator of adipose distribution and function in more than 30 publications [8]. However, BMI mainly reflects overall obesity, and WC varies with the phases of respiration and fullness of the stomach. Moreover, both WHR and WC are based on longitudinal measures that are not very reliable for estimating central obesity in subjects who develop pendulum abdomen, in which the line of the umbilicus falls below the line of the hip. Given this information, all of these indices have certain limitations.

Deposition of fat around the neck is a unique phenomenon that depicts upper body subcutaneous adipose tissue. Measurement of the neck circumference (NC) has been identified as a surrogate marker for determining upper-body subcutaneous fat distribution. It is low-cost, reliable, noninvasive, reproducible and unaffected by phases of respiration or stomach fullness. NC has been proven to be closely associated with other anthropometric parameters (e.g., BMI and WC) and various metabolic risk factors [9–12]. Previous studies also showed that NC or neck fat content is positively correlated with visceral fat content; however, these studies were conducted in HIV-infected individuals, with small sample sizes or in combination with other diseases, limiting the degree to which the findings can be generalized [11, 13, 14]. Here, we aimed to find whether NC is a good indicator for evaluating visceral obesity in the general population by comparing the relationship between NC and the fatness indices expressing visceral fat distribution.

## Methods

### Study population

**Fig. 1:**
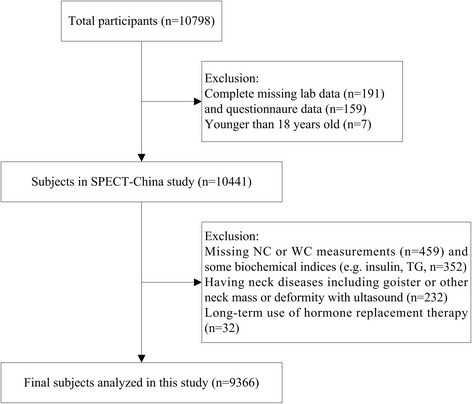
Flowchart of participants selected from SPECT-China

SPECT-China is a cross-sectional survey on the prevalence of metabolic diseases and risk factors in East China (ChiCTRECS-14,005,052, www.chictr.org.cn). The details of the study design have been described previously [15–17]. In brief, the study was performed from February 2014 to December 2015. Twenty-two sites in Shanghai, Jiangxi Province, Zhejiang Province, Jiangsu Province and Anhui Province were selected using a stratified cluster sampling method. Adults aged 18 years and older who were Chinese citizens and had lived at their current residence for 6 months or longer were invited to participate in our study. Those with severe communication problems, with acute illness or who were unwilling to participate were excluded from the study. A total of 10,798 residents participated in this investigation. After exclusion of participants who were completely missing laboratory results (*n* = 191), were missing questionnaire data (*n* = 159), or were younger than 18 years old (*n* = 7), 10,441 subjects were enrolled in the SPECT-China study. Candidates with missing NC or WC measurements (*n* = 459) and some biochemical indices (e.g., insulin or TG levels, *n* = 352) were also excluded. In addition, patients having neck diseases including goiter or other neck masses or deformities identified with ultrasound diagnosis (*n* = 232) or with long-term use of hormone replacement therapy (*n* = 32) were excluded. Finally, a total number of 9366 participants aged 18–93 years were analyzed in this study (Fig. 1).

The study design protocol was approved by the Ethics Committee of Shanghai Ninth People’s Hospital, Shanghai JiaoTong University School of Medicine. All procedures were in accordance with the ethical standards of the responsible committee on human experimentation (institutional and national) and with the Helsinki Declaration of 1975, as revised in 2008. Written informed consents were obtained from all patients.

### Clinical and anthropometric evaluation

At every site, the same trained staff collected all the data according to a standard protocol. Trained interviewers used a questionnaire to collect information on demographic characteristics, medical history and lifestyle risk factors [18]. Current smoking was defined as having smoked at least 100 cigarettes in one’s lifetime and currently smoking cigarettes, and current drinking was defined as alcohol intake more than once per month during the past 12 months [18]. Body weight, height, WC, HC and blood pressure were measured with the use of standard methods as described previously [12, 18]. NC was measured below the cricoid cartilage, and afterwards, at the level of the mid-cervical spine [19]. BMI was calculated as the weight in kilograms divided by the height in meters squared. WHR and WHtR were calculated as waist circumference divided by hip circumference and height, respectively. The VAI, LAP, AVI and Cindex were calculated as follows [6, 7]:

$$ \mathrm{Males}:\mathrm{VAI}=\mathrm{WC}\left(\mathrm{cm}\right)/\left[39.68+1.88\times \mathrm{BMI}\ \left(\mathrm{kg}/{\mathrm{m}}^2\right)\right]\times \left[\mathrm{TG}\left(\mathrm{mmol}/\mathrm{L}\right)/1.03\right]\times \left[1.31/\mathrm{HDL}\ \left(\mathrm{mmol}/\mathrm{L}\right)\right]\ \mathrm{LAP}=\left[\mathrm{WC}\ \left(\mathrm{cm}\right)\hbox{--} 65\right]\times \mathrm{TG}\ \left(\mathrm{mmol}/\mathrm{L}\right) $$

$$ \mathrm{Females}:\mathrm{VAI}=\mathrm{WC}\left(\mathrm{cm}\right)/\left[36.58+1.89\times \mathrm{BMI}\ \left(\mathrm{kg}/{\mathrm{m}}^2\right)\right]\times \left[\mathrm{TG}\ \left(\mathrm{mmol}/\mathrm{L}\right)/0.81\right]\times \left[1.52/\mathrm{HDL}\ \left(\mathrm{mmol}/\mathrm{L}\right)\right]\ \mathrm{LAP}=\left[\mathrm{WC}\ \left(\mathrm{cm}\right)\hbox{--} 58\right]\times \mathrm{TG}\ \left(\mathrm{mmol}/\mathrm{L}\right). $$

$$ \mathrm{For}\ \mathrm{both}\ \mathrm{males}\ \mathrm{and}\ \mathrm{females}:\mathrm{AVI}=\left[2\ \mathrm{cm}\times {\mathrm{WC}}^2{\left(\mathrm{cm}\right)}^2+0.7\ \mathrm{cm}\times {\left(\mathrm{WC}\hbox{--} \mathrm{HC}\right)}^2\right]/1000\ \mathrm{Cindex}=\mathrm{WC}\left(\mathrm{m}\right)/0.109\times \surd \left[\mathrm{weight}\left(\mathrm{kg}\right)/\mathrm{height}\left(\mathrm{m}\right)\right]. $$

### Biochemical measurements

Peripheral venous blood samples were drawn after an overnight fast of at least 8 h. The blood samples for the plasma glucose test were collected into vacuum tubes with the anticoagulant sodium fluoride and centrifuged within 1 h after collection. All blood samples were stored at − 20 °C after collection and then shipped by air within 2–4 h on dry ice to a central laboratory, which was certified by the College of American Pathologists. Glycated hemoglobin (HbA1c) was assessed by high-performance liquid chromatography (MQ-2000PT, China). Fasting plasma glucose (FPG), alanine aminotransferase (ALT) and a lipid profile including total cholesterol (TC), TG, low-density lipoprotein (LDL) and HDL were measured by a BECKMAN COULTER AU 680 (Germany). Insulin was detected by the chemiluminescence method (Abbott i2000 SR, USA). Then, insulin resistance was estimated by the homeostatic model assessment (HOMA-IR) index: [FINS (mIU/L) × FPG (mmol/L)]/22.5.

### Definition of variables

Visceral obesity was defined as a waist circumference ≥ 90 cm in males and ≥ 80 cm in females [18].

### Statistical analysis

Data analyses were performed with the software package SPSS Statistics, Version 22 (IBM Corporation, Armonk, NY, USA). Normally distributed data were expressed as the means ± SD, whereas continuous variables with a skewed distribution were summarized as the median with interquartile range (25%, 75%) and were logarithmically transformed before analysis. To compare the differences between groups, one-way analysis of variance (ANOVA) was used for continuous variables with a Gaussian distribution, and the Kruskal-Wallis test was used for variables with a skewed distribution. Spearman’s correlation coefficient was employed to test the correlations between different variables. The association of NC (independent variable) with fatness indices including WC, WHR, WHtR, VAI, LAP, AVI and Cindex (dependent variables) was assessed by linear regression. Model 1 did not adjust for any factors. Model 2 adjusted for age, smoking, drinking, HbA1c, TC, LDL and SBP. Binary logistic regression analysis was also conducted to explore the association of NC with visceral obesity. Similarly, Model 1 did not adjust for any factors. Model 2 adjusted for age, smoking, drinking, HbA1c, TC, LDL and SBP. A two-sided *P* value < 0.05 was considered statistically significant.

## Results

### Characteristics of study participants

This study recruited 9366 subjects whose mean age was 53.04 ± 13.02 years. Among them, 3938 (42.05%) were male and 5428 (57.95%) were female, with a slight female preponderance (F:M = 1.4:1). The characteristics of the study participants according to the quartile of NC were summarized in Tables 1 and 2. The mean value of NC was 33.52 ± 3.29 cm in the total participants, and men had a mean NC 4.0 cm wider than women (35.84 ± 2.75 vs 31.84 ± 2.54 cm). The quartile ranges of NC in males were ≤ 34, 34–36, 36–38 and > 38 cm. Males with a larger NC had higher values for weight, WC, HC, BMI, WHR, WHtR and blood pressure and higher levels of FPG, HbA1c, FINS, HOMA-IR, TG, TC, LDL and ALT (all *P* < 0.001). The indices (VAI, LAP, AVI and Cindex) assessing visceral fat accumulation also showed a graded increase as NC increased (all *P* < 0.001). At the same time, the quartile ranges for NC in females were ≤ 30, 30–32, 32–33 and > 33 cm. Females with a larger NC also had higher values for metabolic parameters and the fatness indices (all *P* < 0.001). In contrast, the participants with a larger NC displayed lower levels of HDL, both in males and females (all *P* < 0.001).

**Table 1:** Characteristics of study participants according to neck circumference quartiles in males

Male	Neck circumference (cm)				*P*
	≤34	34–36	36–38	> 38	
*N*	1240	1152	903	643	
Age (y)	55.55 ± 13.78	52.95 ± 13.43	53.00 ± 12.23	52.83 ± 12.05	< 0.001
Weight (kg)	61.64 ± 8.22	68.52 ± 7.75	74.69 ± 7.87	81.77 ± 9.71	< 0.001
WC (cm)	77.75 ± 7.91	82.87 ± 7.38	88.12 ± 7.86	93.75 ± 7.79	< 0.001
HC (cm)	89.87 ± 5.47	93.66 ± 5.37	96.91 ± 4.96	100.44 ± 6.23	< 0.001
BMI (kg/m^2^)	22.38 ± 2.60	24.31 ± 2.40	26.20 ± 2.40	28.49 ± 2.86	< 0.001
WHR	0.87 ± 0.07	0.89 ± 0.07	0.91 ± 0.07	0.93 ± 0.07	< 0.001
WHtR	0.47 ± 0.05	0.49 ± 0.05	0.52 ± 0.05	0.55 ± 0.05	< 0.001
SBP (mmHg)	131.78 ± 21.87	132.49 ± 20.08	135.87 ± 19.51	140.60 ± 20.22	< 0.001
DBP (mmHg)	79.64 ± 12.99	81.31 ± 12.80	83.73 ± 12.61	86.76 ± 13.01	< 0.001
FPG (mmol/L)	5.52 ± 1.18	5.59 ± 1.40	5.81 ± 1.64	6.12 ± 1.92	< 0.001
HbA1c (%)	5.49 ± 0.86	5.54 ± 0.99	5.69 ± 1.09	5.90 ± 1.20	< 0.001
FINS (pmol/L)	23.40 (16.30–32.98)	28.30 (19.53–40.08)	35.50 (24.70–49.30)	43.80 (29.90–64.70)	< 0.001
HOMA-IR	0.81 (0.55–1.17)	0.97 (0.67–1.42)	1.27 (0.84–1.80)	1.59 (1.07–2.47)	< 0.001
TG (mmol/L)	1.15 (0.87–1.62)	1.37 (0.98–1.98)	1.66 (1.17–2.53)	1.95 (1.35–2.81)	< 0.001
TC (mmol/L)	5.01 ± 0.89	5.08 ± 1.02	5.25 ± 1.02	5.33 ± 1.10	< 0.001
LDL (mmol/L)	2.93 ± 0.70	3.06 ± 0.79	3.24 ± 0.74	3.24 ± 0.77	< 0.001
HDL (mmol/L)	1.47 ± 0.34	1.36 ± 0.29	1.30 ± 0.28	1.27 ± 0.29	< 0.001
VAI	0.99 (0.67–1.48)	1.30 (0.85–2.03)	1.69 (1.07–2.67)	1.96 (1.32–3.13)	< 0.001
LAP	14.74 (7.04–24.46)	24.13 (14.50–41.14)	39.69 (24.08–62.56)	53.70 (36.04–87.29)	< 0.001
AVI	11.98 (10.65–13.80)	13.86 (12.27–15.50)	15.54 (13.94–17.30)	17.68 (15.86–19.66)	< 0.001
Cindex	43.54 ± 6.41	48.62 ± 6.06	53.82 ± 6.56	59.83 ± 7.42	< 0.001
ALT (U/L)	19 (14–26)	20 (16–28)	23 (17–33)	27 (19–39)	< 0.001

Data were presented as the mean ± SD for continuous variables with a normal distribution and as the median (interquartile range) for continuous variables with a skewed distribution. One–way ANOVA and the Kruskal-Wallis test were used to compare the differences between groups

*WC* waist circumference, *HC* hip circumference, *BMI* body mass index, *WHR* waist-to-hip ratio, *WHtR* waist-to-height ratio, *SBP* systolic blood pressure, *DBP* diastolic blood pressure, *FPG* fasting plasma glucose, *HbA1c* glycated hemoglobin, *FINS* fasting insulin, *HOMA-IR* insulin resistance index, *TG* triglycerides, *TC* total cholesterol, *LDL* low-density lipoprotein, *HDL* high-density lipoprotein, *VAI* visceral adiposity index, *LAP* lipid accumulation product, *AVI* abdominal volume index, *Cindex* conicity index

**Table 2:** Characteristics of study participants according to neck circumference quartiles in females

Female	Neck circumference (cm)				*P*
	≤30	30–32	32–33	> 33	
*N*	1737	1756	650	1285	
Age (y)	50.68 ± 13.68	52.10 ± 12.63	53.06 ± 12.41	55.31 ± 12.04	< 0.001
Weight (kg)	53.03 ± 6.66	59.37 ± 7.19	63.03 ± 7.92	66.68 ± 9.68	< 0.001
WC (cm)	71.12 ± 7.77	76.97 ± 7.62	80.72 ± 7.95	85.89 ± 9.57	< 0.001
HC (cm)	88.23 ± 5.29	92.31 ± 5.81	94.87 ± 6.05	97.98 ± 7.13	< 0.001
BMI (kg/m^2^)	21.85 ± 2.67	24.17 ± 3.03	25.37 ± 3.06	26.93 ± 3.65	< 0.001
WHR	0.81 ± 0.07	0.83 ± 0.07	0.85 ± 0.07	0.88 ± 0.08	< 0.001
WHtR	0.46 ± 0.06	0.49 ± 0.06	0.51 ± 0.06	0.55 ± 0.07	< 0.001
SBP (mmHg)	124.72 ± 21.76	130.30 ± 22.24	131.81 ± 21.88	137.18 ± 21.50	< 0.001
DBP (mmHg)	74.26 ± 12.35	77.54 ± 12.76	79.10 ± 12.82	81.04 ± 13.14	< 0.001
FPG (mmol/L)	5.35 ± 1.10	5.51 ± 1.32	5.53 ± 1.00	6.03 ± 1.73	< 0.001
HbA1c (%)	5.29 ± 0.77	5.43 ± 0.89	5.44 ± 0.77	5.73 ± 1.09	< 0.001
FINS (pmol/L)	28.40 (21.40–38.50)	35.25 (26.13–47.58)	38.40 (27.80–51.53)	44.40 (31.95–63.85)	< 0.001
HOMA-IR	0.96 (0.70–1.32)	1.19 (0.87–1.69)	1.33 (0.95–1.85)	1.60 (1.13–2.48)	< 0.001
TG (mmol/L)	1.06 (0.80–1.45)	1.26 (0.90–1.76)	1.35 (0.96–2.00)	1.49 (1.06–2.12)	< 0.001
TC (mmol/L)	5.08 ± 1.04	5.16 ± 1.05	5.25 ± 1.03	5.25 ± 0.99	< 0.001
LDL (mmol/L)	3.00 ± 0.80	3.10 ± 0.80	3.20 ± 0.83	3.16 ± 0.77	< 0.001
HDL (mmol/L)	1.58 ± 0.31	1.49 ± 0.29	1.45 ± 0.28	1.40 ± 0.28	< 0.001
VAI	1.16 (0.79–1.73)	1.51 (1.00–2.30)	1.70 (1.11–2.67)	1.98 (1.28,3.07)	< 0.001
LAP	12.60 (6.65–22.92)	22.96 (13.44–38.00)	31.28 (18.32–49.30)	42.90 (24.64–66.01)	< 0.001
AVI	10.08 (8.95–11.65)	11.94 (10.45–13.50)	13.05 (11.58–14.91)	14.89 (12.96–17.01)	< 0.001
Cindex	38.13 ± 5.73	43.51 ± 6.10	46.91 ± 6.70	51.43 ± 8.52	< 0.001
ALT (U/L)	14 (11–19)	16 (12–21)	17 (13–22)	18 (14–26)	< 0.001

Data are presented as the mean ± SD for continuous variables with a normal distribution and as the median (interquartile range) for continuous variables with a skewed distribution. ANOVA and the Kruskal-Wallis test were used to compare the differences between groups

*WC* waist circumference, *HC* hip circumference, *BMI* body mass index, *WHR* waist-to-hip ratio, *WHtR* waist-to-height ratio, *SBP* systolic blood pressure, *DBP* diastolic blood pressure, *FPG* fasting plasma glucose, *HbA1c* glycated hemoglobin, *FINS* fasting insulin, *HOMA-IR* insulin resistance index, *TG* triglycerides, *TC* total cholesterol, *LDL* low-density lipoprotein, *HDL* high-density lipoprotein, *VAI* visceral adiposity index, *LAP* lipid accumulation product, *AVI* abdominal volume index, *Cindex* conicity index

### Correlation coefficients for NC

Correlation analysis demonstrated that NC had a positive correlation with all the fatness indices for both overall obesity and visceral obesity, including BMI, WC, WHR, WHtR, VAI, LAP, AVI and Cindex, regardless of sex (all *P* < 0.001). In addition, NC was positively correlated with blood pressure, FPG, HbA1c, FINS, HOMA-IR, TG, TC and LDL but negatively correlated with HDL (all *P* < 0.001) (Table 3).

**Table 3:** Correlation between neck circumference and fatness indices and metabolic parameters by sex

Variables	Male		Female	
	*r*	*P*	*r*	*P*
BMI (kg/m^2^)	0.668	< 0.001	0.569	< 0.001
WC (cm)	0.61	< 0.001	0.584	< 0.001
WHR	0.362	< 0.001	0.382	< 0.001
WHtR	0.531	< 0.001	0.519	< 0.001
VAI	0.382	< 0.001	0.32	< 0.001
LAP	0.557	< 0.001	0.503	< 0.001
AVI	0.617	< 0.001	0.59	< 0.001
Cindex	0.682	< 0.001	0.624	< 0.001
SBP (mmHg)	0.139	< 0.001	0.227	< 0.001
DBP (mmHg)	0.185	< 0.001	0.21	< 0.001
FPG (mmol/L)	0.117	< 0.001	0.23	< 0.001
HbA1c (%)	0.107	< 0.001	0.196	< 0.001
FINS (pmol/L)	0.366	< 0.001	0.331	< 0.001
HOMA-IR	0.375	< 0.001	0.354	< 0.001
TG (mmol/L)	0.334	< 0.001	0.255	< 0.001
TC (mmol/L)	0.114	< 0.001	0.08	< 0.001
LDL (mmol/L)	0.173	< 0.001	0.1	< 0.001
HDL (mmol/L)	−0.262	< 0.001	−0.231	< 0.001

Data were Spearman’s correlation coefficients

### Association of NC with visceral adiposity indices

Table 4 summarized the results of the linear regression models analyzing the association of NC with WC, WHR, WHtR, LnVAI, LnLAP, LnAVI and Cindex. In the base model (Table 4, model 1), a larger NC was associated with higher values on all the visceral adiposity indices in both males and females (all *P* < 0.001). After adjustment for age, smoking, drinking, HbA1c, TC, LDL and SBP, the association was slightly weakened in both sexes but was still highly significant (all *P <* 0.001), and R^2^ was greatly increased in both males and females (Table 4, model 2).

**Table 4:** Association of NC with visceral adiposity indicators: linear regression

Dependent variables	Male				Female			
	β	B (95% CI)	*P*	*R* ^*2*^	β	B (95% CI)	*P*	*R* ^*2*^
WC (model 1)	0.600	2.090 (2.003–2.177)	< 0.001	0.360	0.570	2.224 (2.139–2.310)	< 0.001	0.325
WC (model 2)	0.565	1.974 (1.887–2.061)	< 0.001	0.446	0.483	1.871 (1.795–1.947)	< 0.001	0.509
WHR (model 1)	0.323	0.009 (0.008–0.010)	< 0.001	0.105	0.329	0.010 (0.009–0.011)	< 0.001	0.108
WHR (model 2)	0.304	0.008 (0.007–0.009)	< 0.001	0.208	0.248	0.008 (0.007–0.008)	< 0.001	0.313
WHtR (model 1)	0.526	0.011 (0.010–0.012)	< 0.001	0.276	0.509	0.013 (0.013–0.014)	< 0.001	0.259
WHtR (model 2)	0.504	0.011 (0.010–0.011)	< 0.001	0.414	0.412	0.011 (0.010–0.011)	< 0.001	0.503
LnVAI (model 1)	0.361	0.095 (0.087–0.102)	< 0.001	0.130	0.297	0.076 (0.069–0.082)	< 0.001	0.088
LnVAI (model 2)	0.324	0.085 (0.077–0.093)	< 0.001	0.169	0.221	0.056 (0.050–0.063)	< 0.001	0.218
LnLAP (model 1)	0.521	0.186 (0.176–0.195)	< 0.001	0.271	0.465	0.175 (0.167–0.184)	< 0.001	0.216
LnLAP (model 2)	0.477	0.170 (0.161–0.180)	< 0.001	0.352	0.375	0.141 (0.134–0.149)	< 0.001	0.454
LnAVI (model 1)	0.605	0.049 (0.047–0.051)	< 0.001	0.366	0.574	0.054 (0.052–0.056)	< 0.001	0.329
LnAVI (model 2)	0.570	0.046 (0.044–0.048)	< 0.001	0.455	0.487	0.046 (0.044–0.048)	< 0.001	0.512
Cindex (model 1)	0.675	2.132 (2.059–2.205)	< 0.001	0.456	0.612	2.018 (1.948–2.087)	< 0.001	0.374
Cindex (model 2)	0.634	2.007 (1.933–2.081)	< 0.001	0.512	0.535	1.749 (1.684–1.813)	< 0.001	0.505

R^2^ represented the coefficient of determinationSince VAI, LAP and AVI were non-normally distributed, they were Ln-transformed

Model 1 did not adjust for any factors;

Model 2 adjusted for age, smoking, drinking, HbA1c, TC, LDL and SBP

### Association of NC with visceral obesity

Table 5 demonstrated the results of the logistic regression measuring the association of NC with visceral obesity. In each model, the odds ratio (OR) for visceral obesity increased across NC quartiles (all *P* for trend < 0.001). In the model not adjusting for any factors (Table 5, model 1), compared with the lowest quartile of NC, the OR values for visceral obesity in the highest quartile of NC were 31.00 (95% CI 23.64–40.65; *P* < 0.001) in males and 19.85 (95% CI 16.48–23.92; *P* < 0.001) in females. After adjusting for the potential confounding factors, some OR values increased slightly in both sexes (*P* for trend< 0.001). The OR values for visceral obesity in the highest quartile of NC were 32.34 (95% CI 24.02–43.53; *P* < 0.001) in males and 21.43 (95% CI 17.30–26.55; *P* < 0.001) in females (Table 5, model 2).

**Table 5:** Association of NC with visceral obesity: logistic regression

NC (cm)	Male		NC (cm)	Female	
	Model 1	Model 2		Model 1	Model 2
Q1 (≤34)	1.00 (Ref.)	1.00 (Ref.)	Q1 (≤30)	1.00 (Ref.)	1.00 (Ref.)
Q2 (34–36)	2.73 (2.12–3.53)	2.80 (2.13–3.69)	Q2 (30–32)	3.20 (2.72–3.78)	3.20 (2.66–3.86)
Q3 (36–38)	8.29 (6.47–10.62)	8.19 (6.26–10.71)	Q3 (32–33)	7.63 (6.22–9.36)	8.37 (6.63–10.58)
Q4 (> 38)	31.00 (23.64–40.65)	32.34 (24.02–43.53)	Q4 (> 33)	19.85 (16.48–23.92)	21.43 (17.30–26.55)
*P* for trend	< 0.001	< 0.001	*P* for trend	< 0.001	< 0.001

The data were expressed as the odds ratio (95% confidence interval). *NC* neck circumference

Model 1 did not adjust for any factors;

Model 2 adjusted for age, smoking, drinking, HbA1c, TC, LDL and SBP

## Discussion

We found that NC was highly correlated with all the anthropometric indices of obesity including visceral obesity and overall obesity. After full adjustment for demographic variables and metabolic factors, NC was still significantly associated with visceral obesity and those fatness indices evaluating visceral fat distribution, including WC, WHR, WHtR, VAI, LAP, AVI and Cindex.

The emergence of visceral fat could be interpreted as a specific marker of systemic lipid over-accumulation, expressed by a parallel increase in circulating TG. The excess lipids may be stored in the ectopic sites (e.g., skeletal muscle, liver and pancreatic β cells), where they can cause substantial metabolic disruption [5]. Additionally, visceral adiposity can produce more free fatty acids [5] and secrete a large number of inflammatory cytokines, cells and adipokines, which may play important roles in the occurrence of insulin resistance (IR) and diabetes [20]. Classically, BMI is the most widely used index to measure total adiposity, while WC, WHR and WHtR have been used as surrogate markers for visceral adiposity. In our study, NC is significantly correlated with these fatness indices, which corresponded well with previous studies [9, 11]. A cross-sectional study on elderly Chinese subjects found NC to be highly correlated with BMI and WC [21]. Another study in diabetic individuals found a positive correlation of NC with obesity markers [22]. However, BMI can neither distinguish between fat and lean tissues nor identify the anatomic location or function of distinct fat depots [23]. WC also has a number of limitations. First, the site for measurement of WC varies in different clinical studies [24, 25]. Second, WC is affected by the state of stomach fullness and respiration. Third, it may not be practical for large population studies, especially in cold weather and with heavy clothing [25]. NC has been suggested to be a better indicator for evaluating central obesity compared with other anthropometric indices [26], due to the advantages of being a stable and convenient measurement at an explicit anatomic landmark with little fluctuation related to diet and respiratory conditions. However, these studies were conducted in specific populations or with small sample sizes. The most important advantage of this study is that it is the first study to verify the association between NC and fatness indices in the general Chinese population with a large sample size.

Another interesting finding from our study was that NC, to some extent, might be able to not only identify visceral adipose distribution but also visceral fat function for the general population. The VAI has been identified to be an effective marker for visceral obesity, which can replace visceral CT scanning [27]. In addition, VAI could evaluate visceral adipose function with high sensitivity and specificity. The LAP, a continuous marker, could reflect the combined anatomic and physiologic changes associated with lipid over-accumulation in adults [23]. The AVI was an estimation of the overall abdominal volume that theoretically included both intra-abdominal fat and adipose tissue volume [6]. Cindex, an index of abdominal obesity, was shown to be a sensitive indicator of risk for hyperlipidemia in Western populations [7]. Our results showed that NC was significantly associated with these indices. In addition, many pro-inflammatory molecules secreted by adipocytes are involved in regulating metabolic and immune functions. Previous studies showed a correlation between NC and several adipose cytokines, which suggested a role for NC in reflecting adipose tissue function and whole-body metabolic conditions [9]. Jamar et al. [28] found that plasminogen activator inhibitor 1 (PAI1) is a prothrombotic adipokine involved in the coagulation cascade and fibrinolysis that may increase the risk related to obesity, and NC was shown to be an independent predictor of PAI1 after adjustment for sex and BMI. All these studies indirectly displayed that NC might be able to reflect both visceral fat distribution and function.

We also found that NC was positively associated with blood pressure, FPG, insulin, HOMA-IR, TC, TG and LDL and negatively correlated with HDL. According to Hoebel et al. [29] and others [30, 31], NC can be a useful biomarker of risk factors in metabolic syndrome, such as IR, central obesity, blood pressure, fasting glucose levels and triglycerides. Stabe et al. [25] found that NC was strongly associated with IR. Other research also found that NC could indicate risk factors associated with metabolic syndrome in teenagers [31]. In fact, a previous study had shown that NC individually contributed to the prediction of MS risk factors beyond conventional anthropometric indices such as BMI, WC and WHR [32].

This study has some strengths. First, it is the first study to detect the association between NC and visceral obesity in the general Chinese population. Second, anthropometric measurements were made and questionnaires were administered by the same trained research group with strong quality control. Third, our data source is from a general population, and the sample size was relatively large, so the results may be more reflective of the population as a whole. However, our study also has some limitations. First, being a cross-sectional study, it was difficult to make a causal inference between NC and visceral obesity. Second, it lacked direct measurements of subcutaneous and visceral body fat, such as CT and MRI, which most accurately reflect body fat distribution. However, taking into account the clearly conveyed aim of this study, the absence of such validation does not affect our conclusions. Third, some adipokines that could reflect adipose function were not assayed. Thus, additional studies are needed to verify the association between NC and visceral adipose function. Finally, individuals who practice some sports may have a larger NC. Thus, the information on some special types of sports which might influence the size of NC should be collected in the questionnaire in the future study. Despite these limitations, we were still able to make assumptions regarding the usefulness of NC in diagnosing visceral obesity, especially fat distribution.

## Conclusions

NC was found to be a simple, yet reliable tool for estimating visceral obesity in the general Chinese population. Due to its ease of measurement, it can be considered a first step towards screening for metabolic disorders related to visceral obesity.

## Abbreviations

AVI: Abdominal volume index
BMI: Body mass index
Cindex: Conicity index
DBP: Diastolic blood pressure
FINS: Fasting insulin
FPG: Fasting plasma glucose
HbA1c: Glycated hemoglobin
HC: Hip circumference
HDL: High-density lipoprotein
HOMA-IR: Insulin resistance index
LAP: lipid Accumulation product
LDL: Low-density lipoprotein
NC: Neck circumference
SBP: Systolic blood pressure
TC: Total cholesterol
TG: Triglycerides
VAI: Visceral adiposity index
WC: Waist circumference
WHR: Waist-to-hip ratio
WHtR: Waist-to-height ratio
